# Impact of climate-induced human migration on dengue exposure risk in Africa

**DOI:** 10.21203/rs.3.rs-9235254/v1

**Published:** 2026-04-08

**Authors:** Desalew Meseret Moges, Jenicca Poongavanan, Graeme Dor, Monika Moir, Cheryl Baxter, Sarah Sparrow, Tulio de Oliveira, Moritz U.G. Kraemer, Houriiyah Tegally

**Affiliations:** 1 Centre for Epidemic Response and Innovation (CERI), School for Data Science and Computational Thinking, Stellenbosch University, Stellenbosch 7600, South Africa; 2 Oxford e-Research Centre, Engineering Science, University of Oxford, United Kingdom; 3 KwaZulu-Natal Research Innovation and Sequencing Platform (KRISP), Nelson R. Mandela School of Medicine, University of KwaZulu-Natal, Durban, South Africa; 4 Department of Biology, University of Oxford, United Kingdom; 5 Pandemic Sciences Institute, University of Oxford, United Kingdom

## Abstract

Climate-induced human migration has become an important factor influencing infectious disease dynamics in Africa. Using continent-wide projections of climate-induced migration and population change through 2050 under multiple socio-economic and emissions scenarios, we assess how cross-border and internal movements shift over space and time, and how these shifts reshape population exposure to dengue. By 2050, climate impacts could force roughly 1.1 million people to move across national borders and nearly 75 million to relocate internally. Cross-border migration is projected to occur mainly within Southern Africa, while internal migration is expected to concentrate people in cities, border regions, and areas with favorable climates. These mobility patterns are estimated to increase dengue exposure by shifting populations into areas where transmission is already established. By mid-century, cross-border migration alone could expose up to 340,000 additional people across the continent. Our findings highlight the need to integrate mobility dynamics into early public health planning and preparedness efforts.

Human migration has been part of human history for centuries^1^, but today anthropogenic climate change is a major driver of climate-related population movements^2^. The World Bank estimates that up to 216 million people could be displaced globally by 2050 due to climate-related impacts, with approximately 86 million in Sub-Saharan Africa^3^. At the continental level, up to 113 million people in Africa (about 5% of today’s total population) may be forced to relocate within their country due to climate impacts in the mid-century. The most vulnerable groups are typically rural, agriculture-dependent, poorer households, and individuals exposed to conflict, for whom migration is an adaptive strategy to seek alternative livelihoods^5,6,7^.

Africa’s high vulnerability to climate change stems from the frequent occurrence of extreme weather events such as droughts, floods, and El Niño, compounded by limited adaptive capacity and a heavy reliance on climate-sensitive sectors^8,9,10^. Rising temperatures and more frequent extreme events lead to crop failures, reduced agricultural productivity, and severe water shortages, exacerbating food insecurity and accelerating climate-induced migration^10^. With over 60% of its population reliant on rainfed agriculture^11,12^, even minor climate shifts can cause major livelihood losses, intensifying migration pressures and making the climate–migration nexus a critical issue for both policymakers and researchers. Here, climate-induced human migration refers to the movement of people within their own countries or across international borders, driven directly or indirectly by climate-related environmental changes^10^.

Climate-induced migration can take many forms, ranging from short-term displacement to long-term or permanent relocation, and can be voluntary or forced^6,13^. Migration patterns are often shaped not only by the type and timing of climate events but also by the social, economic, political, and demographic context of affected communities^10^. Slow-onset stressors, such as prolonged droughts or water scarcity, often lead to gradual migration as livelihoods deteriorate, while sudden shocks such as floods or cyclones trigger immediate displacement, sometimes temporarily^6,13,14^. Importantly, climate change rarely acts alone; rather, it interacts with existing social, political, and economic pressures, amplifying pre-existing vulnerabilities within the community. In this way, climate change can act as a “threat multiplier,” intensifying existing risks and driving migration^15,16,17,18^. Given the interacting factors shaping human migration, it is often difficult to establish a linear, causative relationship between anthropogenic climate change and associated migration^19^. This effect is especially evident in regions like Sub-Saharan Africa, where environmental stress often combines with socio-economic challenges to undermine livelihoods, increase health risks, and produce diverse migration patterns.

Climate migration is increasingly recognized as an important social determinant of health because migration influences physical, mental, and social well-being^17,20^. It is also linked to increased risks of infectious disease transmission^21-24^. As people relocate in response to climate stressors, they may encounter unfamiliar ecological contexts, experience overcrowding in temporary or informal settlements, and face disruptions to healthcare access, sanitation, and preventive services^6,11,25,26,27^. These conditions can facilitate infectious disease transmission, strain health systems in destination areas, and increase risks for both migrant and host populations. In addition, pathogens may be introduced into new settings through infected travelers^28^, with subsequent spread influenced by population immunity and local epidemiological conditions within both migrant and host communities^29^.

Dengue virus is one of the rapidly spreading climate-sensitive arboviruses, with its expanding geographic range dramatically increasing population-level exposure^30,31^. Once largely limited to parts of Asia and Latin America, dengue is now endemic in more than 129 countries, placing nearly 4 billion people at risk^28,30,32,33^. Beyond the climatic and environmental changes that are accelerating dengue’s geographic expansion, human migration has added a layer of complexity by reshaping patterns of population exposure, vulnerability, and transmission^34^. As a result, dengue has become a global health concern that requires increased attention and preparedness not only in dengue-endemic countries but also in regions where sustained transmission has historically been absent^35^.

The climate–migration–infectious disease nexus constitutes one of the most urgent yet underexplored dimensions of contemporary global change^36,37,38^. As climate pressures intensify and their impacts deepen, advancing research at this intersection has become increasingly essential. While climate change and human migration are each widely recognized as major public-health challenges^6,36,39,40,41,42^, how climate-driven population movements alter infectious-disease risks remains poorly understood. This gap is particularly critical in Africa, where acute climate vulnerability, rapid demographic expansion, and accelerating human migration interact with growing environmental suitability for key disease vectors. Despite these intersecting pressures, continent-wide assessments of how climate-induced migration affects arboviral transmission are still lacking.

In this study, we examine how climate-induced internal (within-country) and international (cross-border) migration may reshape population exposure to dengue risk across Africa. Specifically, we aim to (1) analyze the spatial and temporal dynamics of climate-induced migration within and between countries under multiple future development and emission scenarios, and (2) quantify how these migration patterns affect dengue exposure risk through mid-century using counterfactual scenarios with and without migration. By integrating projections of future population distributions, climate-driven migration scenarios, and mechanistic dengue transmission models, we present a novel framework that identifies hotspots of climate-induced migration and dengue risk, offering new insights into how migration may reshape dengue exposure and transmission across Africa and inform proactive, migration-aware public health adaptation strategies.

## Cross-border climate migration dynamics in Africa

Using climate-driven migration projections from the African Climate Migration Initiative (ACMI)^43^, we quantified when and where climate-driven migration is expected to occur within and between countries under three shared socioeconomic and emission scenarios: high development–low emissions (SSP1–2.6), high development–high emissions (SSP1–6.0), and low development–high emissions (SSP3–6.0) (Methods). We base our main analysis on the SSP1–6.0 scenario, which offers a realistic, intermediate projection of future climate-migration patterns, while alternative scenarios are presented in the Supplementary Information.

**Fig. 1:**
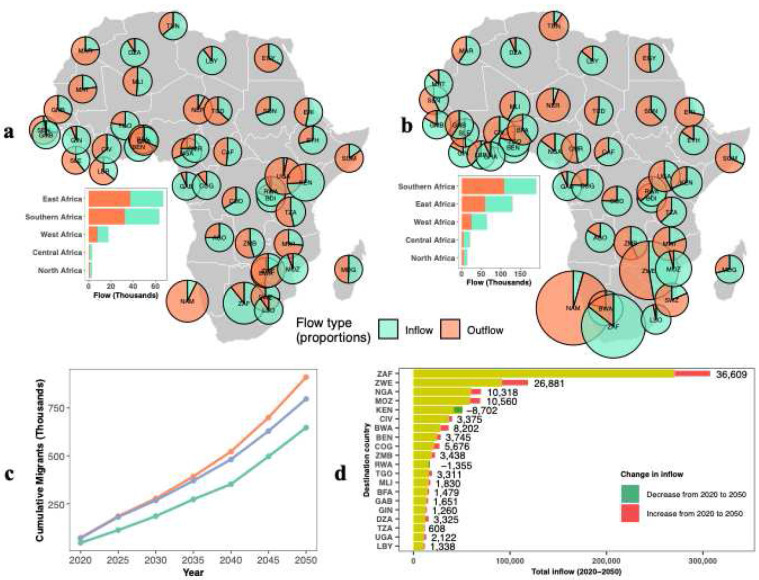
Cross-border climate migration patterns in Africa. **a,b**, Spatial distribution of cross-border migration flows in 2020 **(a)** and 2050 **(b)** under the SSP1–6.0 scenario, where pie charts represent proportions. Bar plots on the left side of each map show the regional distribution of migration inflows and outflows. **c**, Continental trends of cross-border migration over 2020–2050 for three scenarios. **d**, Top 20 destination countries for cross-border migration under SSP1–6.0. Yellow bars indicate cumulative inflows from 2020 to 2050, with numeric labels showing the absolute change between 2020 and 2050 (red indicates an increase, green indicates a decrease). DZA = Algeria; AGO = Angola; BEN = Benin; BWA = Botswana; BFA = Burkina Faso; BDI = Burundi; CMR = Cameroon; CAF = Central African Republic; TCD = Chad; COG = Congo; COD = Democratic Republic of Congo; CIV = Côte d’Ivoire; DJI = Djibouti; EGY = Egypt; GNQ = Equatorial Guinea; ERI = Eritrea; SWZ = Eswatini; ETH = Ethiopia; GAB = Gabon; GMB = Gambia; GHA = Ghana; GIN = Guinea; GNB = Guinea-Bissau; KEN = Kenya; LSO = Lesotho; LBR = Liberia; LBY = Libya; MDG = Madagascar; MWI = Malawi; MLI = Mali; MRT = Mauritania; MAR = Morocco; MOZ = Mozambique; NAM = Namibia; NER = Niger; NGA = Nigeria; RWA = Rwanda; SEN = Senegal; SLE = Sierra Leone; SOM = Somalia; ZAF = South Africa; SDN = Sudan; TZA = Tanzania; TGO = Togo; TUN = Tunisia; UGA = Uganda; ZMB = Zambia; ZWE = Zimbabwe.

Our results reveal that climate-induced cross-border migration is projected to increase steadily from 2020 to 2050 across all African countries and scenarios (Fig. 1a-c; Supplementary Fig. 1). At the continental scale, while all scenarios show large increases in migration over time, SSP1–6.0 reveals consistently higher migration inflows throughout the period, increasing from 71,957 in 2020 to 210,705 in 2050 and resulting in the largest cumulative total of approximately 1.1 million people by 2050 (Fig. 1c). SSP3–6.0 follows an intermediate trajectory, with inflows growing from 71,071 to 167,572 people and a cumulative total of 796,980 by mid-century, compared to 0.64 million under SSP1–2.6. This contrast highlights the strong sensitivity of cross-border migration to emissions trajectories, even under relatively optimistic development pathways.

Climate-induced cross-border migration in Africa shows marked regional heterogeneity. East and Southern Africa experience the largest flows and the strongest shifts between 2020 and 2050, while most of West and Central Africa exhibit comparatively fewer migrations (Fig. 1a,b). East Africa is projected to have rising inflows from 29,000 to 69,000, but overall net migrations are small (outflows are projected to change from 38,000 to 61,000 between 2020 and 2050). In Southern Africa, inflows are projected to nearly triple from 31,000 to 82,000, while outflows are projected to increase from 33,000 to 110,000, indicating growing net out-migration. By mid-century, Southern Africa will emerge as the continent’s migration hub, driven by major outflows from Namibia and inflows to South Africa.

Cumulative projected climate-induced migration inflows from 2020 to 2050 (Fig. 1d; Supplementary Fig. 1e,f) show that Southern and Eastern Africa are the main destinations across all scenarios. South Africa receives the highest number of migrants, with a total inflow of 270,925 and the largest increase (36,609) over this period. Other nearby countries also see fast growth between 2020 and 2050, especially Zimbabwe, where inflows rise more than tenfold from 1,932 to 28,813, and Botswana, where inflows increase more than fourfold from 2,546 to 10,748, pointing to a strong Southward shift in migration. In contrast, Kenya and Rwanda will receive fewer migrants by 2050, suggesting they are becoming transit or source countries rather than destinations within East Africa. Overall, most climate-induced migration occurs between neighboring countries (Supplementary Fig. 2), with major routes from Namibia, Zimbabwe, and Botswana to South Africa, and from Malawi and Zimbabwe to Mozambique. These patterns show that short-distance cross-border movement plays a central role in shaping climate-driven migration in Africa.

## Internal climate migration dynamics in Africa

To assess climate-induced migration within African countries, we used internal migration projections from the ACMI^44^ and applied the same scenarios and methods used in the cross-border analysis (Methods). For cross-border migration, our focus was on inflows because they influence the spread of infectious diseases in destination areas, whereas the internal migration assessment considers inflows and outflows symmetrically.

**Fig. 2:**
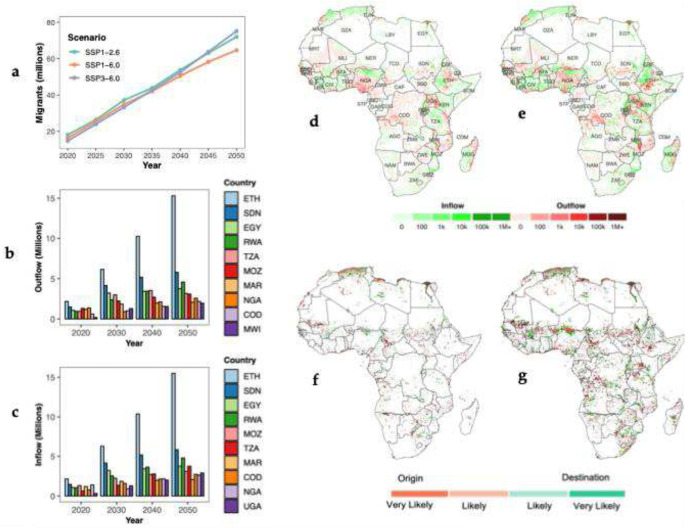
Climate-induced internal migration patterns in Africa. **a,** Continental trends of internal climate migration between 2020 and 2050 under three climate and socio-economic scenarios. **b,c,** Top 10 countries with the greatest internal migration outflows (**b**) and inflows (**c**) between 2020 and 2050 under SSP1-6.0. **d,e,** Spatial distribution of internal climate migration in 2020 (**d**) and 2050 (**e**) under SSP1–6.0. **f,g,** Internal climate migration hotspots in 2020 (**f**) and 2050 (**g**). *Likely* hotspots represent locations where population differences fall within the top 5th percentile (positive or negative) in 2 of the 4 model scenarios, while *very likely* hotspots represent locations where this threshold is met in 3 or 4 of the 4 scenarios. Country codes are shown in Fig. 1.

Our scenario-based projections indicate that internal climate migration will increasingly reshape population distributions across Africa over the coming decades, although the scale of these changes varies by region (Fig 2). Migration patterns remain generally similar across scenarios until the early 2040s (Fig. 2a; Supplementary Fig. 3), after which projections under SSP3–6.0 show the highest levels of internal migration, followed closely by SSP1–2.6. Under the moderate scenario (SSP1–6.0), internal migration is projected to reach approximately 65 million people by mid-century, compared with around 75 million under SSP3–6.0 and 72 million under SSP1–2.6. Ethiopia shows the highest levels of internal migration, with both inflows and outflows projected to be around 16 million people by 2050 (Fig. 2b,c). Other countries expected to experience substantial internal migration include Sudan (~6 million) and Rwanda (~5 million). Egypt, Mozambique, and Tanzania may also exhibit notable internal migration levels, each reaching roughly 3–4 million.

The spatial distribution of internal migration (Fig. 2d,e) indicates that, although migration patterns remain broadly homogeneous over time, the number of people moving is projected to increase substantially by mid-century. The most noticeable increases in movement are projected in Eastern and Western Africa, where both inflows and outflows intensify substantially. By 2050, distinct migration corridors are likely to emerge along major border areas, including the Nigeria–Niger border, the Malawi–Mozambique border, and the Ethiopia–Somalia border. Cities are also expected to function as key hubs within these migration networks. Urban centers such as Khartoum, Maputo, and Kigali are projected to attract migrants from climate-stressed rural areas, while major coastal cities like Lagos, Abidjan, and Accra may see significant outflows due to climate-related hazards.

We also analysed internal climate migration using climate migration hotspots, defined as areas projected to experience substantial population changes across multiple scenarios that incorporate climate change impacts, relative to projections that exclude climate influences^4,44^. Such areas are projected to emerge across much of Africa and are characterized by large population shifts in both migration origin and destination areas (Fig. 2f,g). Identifying migration hotspots is critical for pinpointing regions where climate-driven internal migration is likely to reshape settlement patterns, strain resources, and inform policy planning over the coming decades. By 2050, Northern and Central Ethiopia, the Niger–Nigeria border region, coastal areas of Algeria and Egypt, and the Uganda–Kenya border are projected to emerge as key climate migration hotspots.

## Cross-border climate migration and dengue exposure risk in Africa

We quantified the impact of cross-border migration on dengue exposure risk at the national level by overlaying a fixed 2020 dengue transmission potential map, derived from climate-driven mechanistic models incorporating temperature- and humidity-dependent effects on mosquito biology^30^, with population and migration projections from 2020 to 2050 at five-year intervals. Because dengue-suitable areas are expected to expand under future warming^28^, these estimates represent a conservative assessment of migration-related exposure. By comparing baseline exposure (population at risk before migration) with post-migration exposure, we isolated the contribution of climate-induced human migration to both absolute and relative changes in dengue risk (Methods). To further contextualize the migration-related risk patterns, we also compared the projected exposure risks with current dengue exposure risk levels reported by the Centers for Disease Control and Prevention (CDC)^45^.

**Fig. 3.**
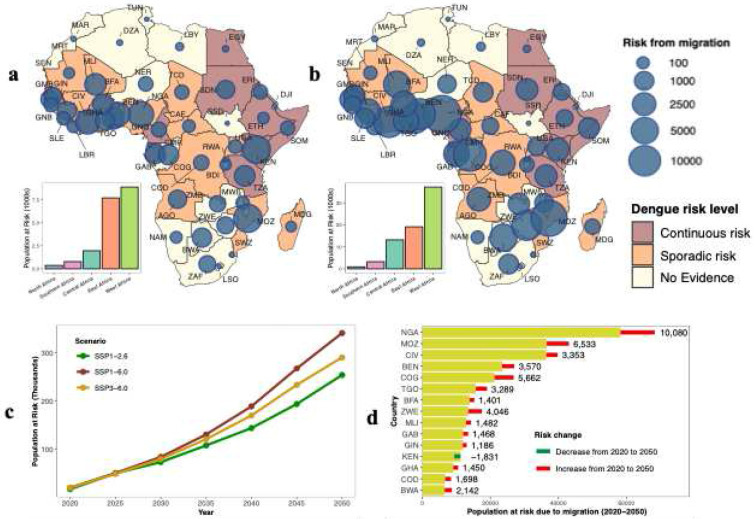
Cross-border climate migration impact on dengue transmission in Africa. **a,b,** Spatial distribution of populations at risk of dengue transmission under climate-induced migration in 2020 (**a**) and 2050 (**b**). Bubble size indicates the population counts at risk attributed to climate-induced migration, while background choropleth shading represents the current dengue risk level according to the Centers for Disease Control and Prevention. Bar plots on the left side of the maps illustrate the regional distribution of dengue risk resulting from cross-border migration. **c,** Total projected continental population at risk of dengue from 2020 to 2050 under different scenarios. **d,** Projected cumulative population at risk from 2020 to 2050 for the top 15 countries. Yellow bars show total populations at risk, with red segments representing increases and green segments representing decreases; absolute changes are labeled.

Our results indicate that climate-induced cross-border migration is projected to substantially alter dengue exposure patterns across Africa under all future scenarios considered (Fig. 3; Supplementary Figs.4-7). Although the number of people projected to be exposed to dengue transmission risk increases across all scenarios over the study period, the largest rise is expected under the SSP1–6.0 scenario (Fig. 3c). At the continental scale, the projected population at risk of dengue infection due to cross-border migration under SSP1–6.0 reaches approximately 340,000 people by 2050, compared with about 290,000 people under SSP3–6.0.

Regionally, cross-border migration substantially increases dengue exposure in West and East Africa through mid-century (Fig. 3a,b, bar plots; Supplementary Fig. 7). However, the projected rate of increase may slow in East Africa by 2050 relative to 2020, whereas West Africa may exhibit a steeper rise. In Southern Africa, countries such as Botswana and South Africa experience the largest relative increases in population exposed to dengue due to migration. Although these countries currently have negligible dengue risk or no documented local transmission, they receive migrants from higher-risk regions (Supplementary Fig. 6), increasing the likelihood of dengue introduction and the establishment of new transmission hotspots. In contrast, migration has minimal impact on dengue exposure in North Africa, where dengue transmission has historically been absent or rare.

Countries with large populations, such as Nigeria, Côte d’Ivoire, Kenya, and the Democratic Republic of Congo, exhibit large absolute increases in the number of people at risk of dengue exposure due to migration (Supplementary Fig. 4c,d). In these countries, which already experience endemic or sporadic dengue risk, migration can further exacerbate the population at risk and may intensify public health burdens. We also observe large absolute and proportional increases in populations exposed to dengue in smaller countries such as Benin, Côte d’Ivoire, Mozambique, and Gabon (Supplementary Fig. 4c,d; Supplementary Fig. 5), where dengue is already endemic or occurs sporadically. This suggests that even relatively modest increases in migrant populations could substantially elevate transmission risk and facilitate local outbreaks.

When considering the cumulative population at risk over the entire study period (Fig. 3d, Supplementary Fig. 4e), most countries experience a substantial increase in dengue exposure driven by migration. Kenya is a notable exception, showing a decline in its cumulative population at risk. This contrast highlights the pronounced spatial heterogeneity in the influence of cross-border migration on dengue risk across Africa.

## Internal climate migration and dengue transmission in Africa

We quantified the impact of internal climate migration on dengue transmission potential at the pixel level by combining gridded dengue transmission suitability, population projections, and projected internal migration flows, all harmonized to a common 10 km resolution (Methods). Comparing baseline and post-migration exposure allowed us to isolate the contribution of climate-induced migration to changes in dengue risk. Grid-level risk estimates were further aggregated to the national and regional scales to evaluate broader patterns of dengue exposure.

**Fig. 4:**
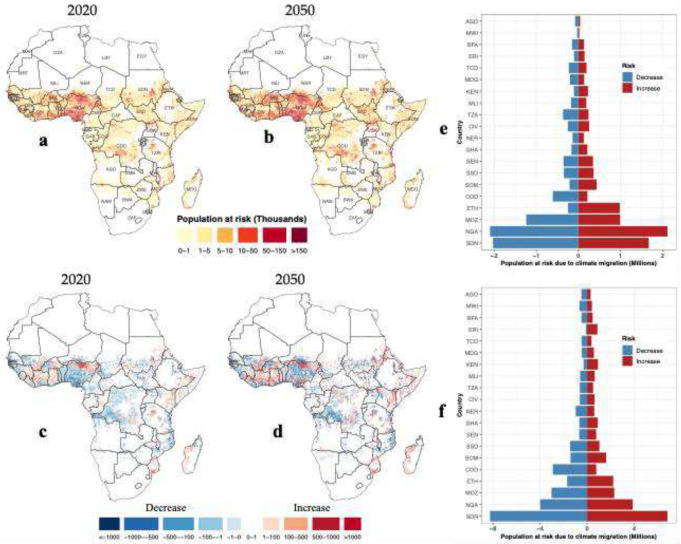
Internal climate migration impact on dengue transmission in Africa. **a,b**, Spatial distribution of total populations at risk of dengue with internal migration in 2020 (**a**) and 2050 (**b**). **c,d**, Change in population at risk of dengue due to migration in 2020 (**c**) and 2050 (**d**), where blue shading indicates decreased exposure and red shading indicates increased exposure. **e,f**, Population change in dengue risk due to migration for the top 20 most affected countries in 2020 (**e**) and 2050 (**f**). All panels depict the SSP1–6.0 scenario. Country codes are shown in Fig. 1.

Our results show that internal climate migration could concentrate dengue risk in a few hotspots. While the overall spatial pattern of population exposure to transmission remains broadly similar over the study period, it shows substantial expansion toward mid-century under all scenarios (Fig. 4; Supplementary Fig. 8). At the continental scale, by 2050, the population exposed to dengue transmission with internal climate migration is projected to exceed 935 million people under SSP1–6.0.

Countries with large populations, such as Nigeria, the Democratic Republic of Congo, and Sudan, consistently bear the greatest burden. In Nigeria alone, the population at risk is projected to increase from approximately 190 million in 2020 to over 317 million by 2050 (Fig. 4a,b) under the conservative dengue risk model scenario. Other countries, including Ghana, Tanzania, Burkina Faso, and Côte d’Ivoire, remain at high risk throughout the study period, while new hotspots emerge by mid-century in countries such as Niger and South Sudan. High population exposure under the current dengue transmission landscape is also evident in several border regions, particularly along the Nigeria–Niger, Cameroon–Nigeria, and Uganda–South Sudan corridors. Moreover, dengue hotspots closely coincide with densely populated areas, with many urban centers situated within transmission-suitable zones (Supplementary Fig. 9).

By isolating the effect of migration, our analysis demonstrates that internal climate migration reshapes dengue transmission risk unevenly across Africa, at both grid- and country-level scales (Fig. 4c-f). In 2020, countries such as the Democratic Republic of Congo, Sudan, Morocco, and Mozambique showed decreases in risk, while parts of East Africa, such as Somalia, Eritrea, and Ethiopia, emerged as early hotspots. By 2050, these patterns become more pronounced, with large increases in risk in Ethiopia, Somalia, Kenya, and Cameroon, while decreases persist in the Democratic Republic of Congo and Morocco across all scenarios (Fig. 4e,f; Supplementary Fig. 8i-l).

## Discussion

Our study provides a continent-wide analysis of how climate-induced internal and cross-border migration may reshape dengue exposure and transmission risk across Africa. By combining migration projections and population estimates with dengue transmission potential data under different climate and socio-economic scenarios through mid-century, we reveal a mechanism that has received relatively limited attention: climate-driven human migration can fundamentally alter the geography of dengue exposure risk. Migration may establish new epidemiological connections between regions, alter the suitability of seasonal transmission in destination areas, and amplify transmission pathways, effects that are often not captured by models that exclude human mobility.

Climate-induced migration across Africa is projected to increase gradually from 2020 to mid-century under all scenarios, although migration levels in 2020 were generally low, likely reflecting COVID–19–related migration restrictions^46^. Migration responses to climate impacts are strongly context dependent. Cross-border migration is highest under SSP1–6.0, whereas SSP3–6.0 is characterized by high internal but limited cross-border movement, reflecting differences in development levels and adaptive capacity that shape how populations respond to climate stressors^47,48,49^. The ACMI migration model assumes that cross-border migration is a function of financial capacity, whereby people in comparatively richer countries are better able to afford the costs of international movement, while people in poorer countries tend to migrate only short distances^50^. This highlights that climate-induced cross-border migration arises not only from environmental stress but also from individual resources that shape people’s ability to move^10,15,51^. Consequently, future climate-driven migration in Africa will be strongly shaped by development trajectories and emissions pathways, underscoring the interconnected nature of climate impacts, socioeconomic resilience, and population migration^9,51,52,53^.

Regionally, climate-induced cross-border migration is most pronounced in Southern Africa, followed by Eastern Africa, identifying these areas as major migration hotspots. Southern Africa is characterized by strong destination–origin contrasts, with South Africa attracting large inflows due to its relatively strong economy and regional employment opportunities^9,54^, while neighboring countries such as Namibia experience substantial out-migration driven by climate extremes, declining water availability and crop yields, limited adaptive capacity, and increasing drought risk under declining precipitation^55,56^. Climate impacts alone are projected to generate annual economic losses of up to 5% of Namibia’s national economy^57^.

East Africa exhibits more complex migration dynamics. Countries like Zimbabwe highlight this complexity through their dual roles as both substantial sources of outflow and important destinations of cross-border migration. Severe exposure to extreme climate events, such as flooding and drought, combined with low adaptive capacity, drives substantial out-migration, while regional economic linkages and established migration networks attract inflows, facilitating multi-step and circular movements with important implications for infectious-disease transmission. By contrast, relatively stable migration patterns in West Africa and declining inflows in parts of East Africa highlight that climate vulnerability alone does not determine migration outcomes; adaptive capacity, population density, and economic opportunity play critical mediating roles^5,15,48^. We also observe that cross-border migration is generally concentrated between neighboring countries. For example, more than half of the projected out-migration from Namibia and Zimbabwe is directed toward South Africa, facilitated by geographic proximity and long-standing regional migration pathways.

Climate-induced migration in Africa occurs predominantly within the country rather than across borders. Our findings show that all African countries are projected to experience internal migration in the coming decades, with magnitudes varying by country, time, socioeconomic conditions, and emission scenarios. By 2050, internal climate-induced migration under SSP3–6.0 is projected to reach approximately 75 million people across the continent and, accounting for existing uncertainties, it could rise to as many as 113 million^4^. Internal migration is primarily driven by recurring climate-related stresses, including droughts, floods, livestock losses, crop failures, pasture shortages, chronic water scarcity, and declining agricultural productivity, all of which intensify food insecurity^10,58,59,60^. Ethiopia demonstrates this dynamic, having experienced severe drought over the past four decades, including four consecutive failed rainy seasons since 2020, resulting in substantial livestock losses^59^. Sudden-onset events such as flooding further displace populations, particularly in low-income urban settlements that are highly exposed to water-borne diseases and malaria^10^. Likewise, pastoralist communities often migrate in search of water and grazing resources^4,61^. In East Africa, pastoral regions in Ethiopia, Kenya, Sudan, and Rwanda are projected to experience high levels of population movement by 2050 due to climate stressors. These pressures are likely to intensify existing challenges faced by pastoral systems across Africa, which are already affected by increasing precipitation variability^4,61^.

Our results show that African borderland regions are emerging as major hotspots of climate-driven internal migration. By 2050, several border corridors, including Nigeria–Niger, Somalia–Kenya, Ethiopia–Sudan, Ethiopia–Somalia, Malawi–Mozambique, and the Democratic Republic of the Congo–Uganda, are projected to experience large-scale population movements. Many of these areas already host large numbers of refugees and internally displaced people^4^, and additional climate-related migration will place even greater pressure on health services, food systems, and fragile local governance^62^. At the same time, climate-induced rural-to-urban migration is likely to push more people into informal settlements that are prone to recurring floods, landslides, sea-level rise, and other hazards. Combined, these patterns increase both the likelihood and the geographic reach of arboviral diseases like dengue across border regions. In particular, border regions with frequent short-term movements can create continuous population mixing, leading to the repeated reintroduction of infectious diseases on both sides of the border and sustaining transmission chains over time.

Climate-induced migration shapes dengue transmission risks for both migrant and host communities through multiple and interacting pathways. Population movement can introduce dengue into new areas or expose migrants to circulating virus serotypes for which they have little or no immunity^23,63^. Our findings show that climate-migration hotspots, particularly border regions and cities, are projected to become dengue transmission hotspots. These areas often have insecure housing, unsafe water-storage practices, and limited access to health care, conditions that increase human contact with *Aedes* mosquitoes and increase susceptibility to transmission risk^38,41,53^. When migrants resettle in areas with different mosquito suitability, dominant serotypes, or seasonal transmission patterns, their risk shifts across space and time in ways that cannot be explained by climate exposure alone^34^. Many migrants also return home without receiving pre-travel health guidance, further facilitating the spread of infection^63^. In rapidly growing cities and border regions, dense living conditions and high levels of short-term movement amplify dengue transmission by increasing both human-to-human and vector-to-human contact^23,33,34^.

The effects of migration on dengue dynamics differ between internal and cross-border migration, yet the two processes remain closely interconnected. Our analysis indicates that internal migration tends to concentrate people in a small number of climatically suitable urban centers, creating amplifier zones where dengue spreads quickly, increasing risk in destinations while reducing it in areas experiencing outmigration^31,64,65^. In contrast, cross-border migration spreads dengue along transport routes and into border towns, where high migration, overcrowding, and limited access to pre-travel health information facilitate repeated viral introduction^3^. These patterns underscore the need for targeted investments in borderland regions to improve the delivery of essential services, expand access to early-warning systems, and establish routine entomological surveillance.

We found that when current dengue risk levels^45^ are considered, climate-driven migration in Africa redistributes populations across exposure levels, affecting both dengue-endemic countries and those with little or no historical risk in different ways. When combined with expanding climate suitability, migration from dengue-endemic regions such as Ethiopia, Kenya, and Tanzania to historically low-risk countries like South Africa and Botswana (Supplementary Fig. 6) could result in sharp relative increases in transmission potential, even with modest migrant inflows^34^. In such conditions, key exposure risks include the introduction of new viral lineages, the emergence of small yet intense transmission clusters, and underdetection due to limited local experience with arboviral disease^66^. Public health responses should therefore prioritize early detection and rapid response through event-based surveillance and rapid genomic tracking, with particular focus on border areas and transport corridors^67,68^. Targeted health communication for migrant communities, port-of-entry screening, and pre-travel vaccination can also reduce the likelihood of introduction and local transmission^27,42^. Unless controlled immediately, the introduction of dengue can trigger rapid transmission and explosive outbreaks due to its fast spread. A One Health approach that integrates human surveillance, vector monitoring, and environmental management is critical for early and effective prevention^69,70^.

In contrast, climate-driven migration inflows in countries with established or recurrent dengue risk can intensify exposure by adding population pressure to already highly favorable transmission environments. These inflows amplify health-system strain, increase outbreak frequency, and can intensify disease severity. This pattern is especially pronounced in West Africa, where large populations, widespread *Aedes* vector presence, and ongoing viral circulation are already well documented^71^. Over time, this dynamic can reinforce an endemic equilibrium in which dengue transmission is maintained primarily by local environmental and social conditions rather than by new introductions alone. In such settings, public-health strategies must extend beyond routine surveillance. With climate migration adding new population pressures, dengue-endemic countries need stronger vector control, broader access to molecular and serological diagnostics, and continuous community-level surveillance supported by robust early-warning systems^70,72,73^.

Despite the contributions of our study, we acknowledge some limitations. First, climate-induced migration projections are inherently uncertain and scenario- dependent, meaning they cannot fully reflect real-world migration dynamics^74,75^. The Bayesian hierarchical framework used in the ACMI projections is particularly sensitive to socioeconomic development pathways^75^, making it difficult to isolate the influence of climate alone on future migration patterns. Second, the migration datasets exclude African island states, likely underrepresenting coastal and island settings where *Aedes* vectors, human movement, and transport connectivity may exacerbate dengue transmission risk. Third, keeping dengue transmission potential fixed at 2020 levels provides a consistent baseline for comparing exposure across scenarios. However, this simplification limits our ability to assess how climate-driven changes in vector suitability may evolve through 2050. Additional limitations include the coarse spatial and temporal resolution of available data, particularly for internal migration (~15km), which may mask important local dengue transmission dynamics. Moreover, our analysis does not incorporate variation in immunity, vector control efforts, or health system capacity, underscoring the need for future research that integrates these critical determinants of dengue risk.

Overall, our findings underscore that climate-induced migration in Africa is likely to intensify as rising temperatures, droughts, extreme rainfall, and ecosystem degradation increasingly undermine climate-sensitive livelihoods. Supporting the well-being of migrating populations will require coordinated planning to ensure equitable access to resources and services in both origin and destination areas. At the same time, climate-driven population movement will reshape dengue transmission by intersecting with the expansion of vector habitats, heightening epidemiological risks. Strengthening diagnostic and surveillance capacity, particularly in emerging high-risk regions, is therefore essential. Integrated strategies linking climate adaptation, migration planning, and infectious disease preparedness are critical to safeguarding public health in a changing climate.

## Methods

### Climate-induced migration data

We used climate-induced migration projections from the Africa Climate Mobility Initiative (ACMI), covering both cross-border (bilateral) and internal (within-country) population movements. Cross-border projections, developed by the Center for Integrated Earth System Information (CIESIN) and the Global Centre for Climate Mobility (GCCM)^43^, estimate international migration flows among 46 mainland African countries at five-year intervals from 2015 to 2050. On the other hand, internal migration projections, produced by the CUNY Institute for Demographic Research (CIDR) in collaboration with CIESIN and GCCM^44^, capture within-country population mobility on a 7.5 arc-minute (~15km) grid at five-year intervals from 2020 to 2050. To ensure consistency across datasets, analyses focused on the 2020–2050 period under three contrasting Shared Socioeconomic Pathway–Representative Concentration Pathway (SSP–RCP) combinations: SSP1–2.6, SSP1–6.0, SSP3–6.0, with primary emphasis on SSP1–6.0 as a balanced representation of future climate–migration dynamics.

### Population data

Population counts used to estimate dengue exposure relative to the migrating population were obtained from a global gridded dataset at 30-arc-second (~1 km) resolution^76^. Based on the WorldPop dataset, SSP population projections, and other related covariates, we provide a range of future population estimates from 2020 to 2100 at 5-year intervals. Validation against WorldPop at both grid-cell and subnational levels confirms the reliability and accuracy of these projections^76^. For this study, we used population estimates for 2020–2050 under SSP1 (sustainable development) and SSP3 (fragmented development) to align with available internal and cross-border migration data.

### Dengue transmission risk data

We used a high-resolution (~10 km^2^) gridded dataset of dengue transmission potential (IndexP; Supplementary Fig. 10a) derived from mechanistic, climate-driven models developed by Nakase *et al.* (ref.^30^). These models quantify the intrinsic suitability for dengue transmission by *Aedes* mosquitoes by incorporating temperature- and humidity-dependent effects on mosquito survival, biting behavior, viral development, and vector competence. Using this framework, transmission potential was estimated for the year 2020, resulting in spatially continuous gridded surfaces of dengue suitability.

To isolate the effects of human migration, we used a fixed 2020 Index P surface and classified grid cells with IndexP >0.5 as transmission-risk areas (Supplementary Fig. 10b). This threshold remained constant across all years and scenarios, while population counts and migration projections varied. Using a stationary climate suitability baseline allows us to attribute changes in the population at risk solely to migration dynamics and shifts in population distributions, rather than to climate-driven changes in vector suitability. This approach, commonly used in climate–health studies^77^, avoids mixing multiple drivers and helps clarify the independent role of migration in dengue exposure. Since the IndexP threshold marks areas with established climatic suitability for *Aedes aegypti*, the 2020 risk surface offers a stable, epidemiologically meaningful reference for monitoring how population movements alter exposure patterns over time.

To distinguish dengue risk areas from non-risk areas, we converted the Index P into a binary classification. Each grid cell’s continuous Index P value was dichotomized using the threshold:

$$R_{i,j}=\{1,if\;D_{i,j}>\tau ,0,otherwise$$

where $R_{i,t}$ denotes the binary dengue risk status at the grid cell (j), $D_{i,j}$ represents the underlying Index P value, and τ=0.5 is the threshold used to designate conditions as suitable (risk) or unsuitable (non-risk) for dengue transmission.

### Quantifying impacts of cross-border climate migration

To evaluate how climate-induced cross-border migration affects dengue transmission risk, we integrated national migration inflows with spatially detailed dengue suitability and population forecasts. Dengue transmission potential was summarized at the national level by averaging raster cells within each country’s administrative boundaries, creating a consistent baseline of transmission risk over time while maintaining spatial variation at the country scale.

For each 5-year interval from 2020 to 2050, the baseline (without migration) population at risk (PAR) was estimated by overlaying the static dengue suitability surface (2020 Index P) with projected population totals using the following formula:

$${PAR}_{i,t}^{base}=P_{i,t}\times R_{i}$$

where ${PAR}_{i,t}^{base}$ is the population at risk in the country i at year t without migration, $P_{i,t}$ is the total population in the country i at year t, and $R_{i}$ is the proportion of the population residing in dengue-risk areas.

A grid cell was considered at risk if the Index P value exceeded 0.5. Applying this threshold consistently across all years and scenarios ensures comparability in assessing the population at risk, regardless of migration.

To quantify the effect of cross-border migration, migration inflows were assumed to carry the same dengue risk profile as the resident population. The population at risk after migration (risk with migration) was calculated as:

$${PAR}_{i,t}^{post}={PAR}_{i,t}^{base}+(M_{i,t}\times R_{i})$$

where ${PAR}_{i,t}^{post}$ is the adjusted population at risk, including migrants, and $M_{i,t}$ is the number of migrants entering the country i a year t. This assumption allows for a first-order estimation of the additional exposure attributable to human migration, while preserving the relative distribution of risk across the destination country.

Then, we computed the impact of migration on dengue exposure using both absolute and relative metrics:

$$\Delta PAR_{i,t}={PAR}_{i,t}^{post}-{PAR}_{i,t}^{base}\;\%\Delta PAR_{i,t}=100\times \frac{\Delta PAR_{i,t}}{{PAR}_{i,t}^{base}}$$

where $\Delta PAR_{i,t}$ captures the absolute increase in the number of people exposed due to migration, while $\%\Delta PAR_{i,t}$ expresses this change relative to the baseline exposure. Both metrics were evaluated at each time point and cumulatively to capture both incremental and long-term effects.

By integrating a fixed dengue transmission potential surface with dynamically changing population and migration projections, our framework isolates how cross-border movements influence changes in exposure, capturing the interactions among climate, demographics, and human migration. Combining grid-level estimates into national and regional scales enables assessment of country-level vulnerability and shows how population movements collectively shift risk across the continent.

### Quantifying impacts of internal climate migration

To evaluate how internal climate migration impacts dengue transmission risk, we combined gridded population projections, dengue transmission potential, and predicted internal migration flows. Population data (~1 km) was downscaled, and migration projections (~15 km at the equator) were upscaled to a 10 km resolution to align with the dengue suitability grid. Dengue risk was represented as a binary raster $\left(R_{i,j}\right)$, where grid cells with Index P > 0.5 were considered suitable for transmission. Then, PAR at the grid level without migration was calculated for each cell (j) and time t as:

$${PAR}_{i,j,t}^{base}=P_{i,j,t}\times R_{i,j}$$

where ${PAR}_{i,j,t}^{base}$ is the number of people exposed to dengue, $P_{i,j,t}$ is the SSP-aligned population at the grid cell, and $R_{i,j}$ is the binary dengue risk mask.

Next, to incorporate climate-induced internal migration, migration flows $M_{i,j,t,s}$ for the scenario s were added to each grid cell, assuming that migrants are immediately exposed to the local transmission risk. The migration-adjusted population at risk was calculated as:

$${PAR}_{i,j,t,s}^{post}=(P_{i,j,t}+M_{i,j,t,s})\times R_{i,j}$$

Absolute changes in population at risk due to migration were then quantified as:

$$\Delta PAR_{i,j,t,s}={PAR}_{i,j,t,s}^{post}-{PAR}_{i,j,t}^{base}$$

Relative changes (percentage) were expressed as:

$$\%\Delta PAR_{i,j,t,s}=100\times \frac{\Delta PAR_{i,j,t,s}}{{PAR}_{i,j,t}^{base}}$$

Grid-level PAR estimates were then aggregated to the national and regional scales to evaluate broader patterns of dengue exposure. This approach allows quantification of how internal migration redistributes and amplifies dengue risk, highlighting emerging hotspots and regions where population movements may exacerbate existing transmission vulnerability. Regional classifications of African countries often differ across documents; here, we follow the classification adopted in the African Climate Mobility Report^4^ (Supplementary Fig. 11).

In assessing population exposure to dengue risk driven by climate-related migration, we used absolute changes in the number of people at risk rather than population-normalized rates (e.g., cases per 100,000). Because climate-induced migration flows are very small relative to national population sizes, normalizing by population yields tiny values that can obscure meaningful changes and make results harder to interpret. Using absolute numbers instead better captures how many people are newly exposed to dengue risk and provides information that is easier to understand and more useful for public-health planning, resource allocation, and policy decisions.

## Supplementary Material

This is a list of supplementary files associated with this preprint. Click to download.

• Supplementarymaterial.docx

## Data Availability

The data that support the findings of this study are publicly available. Climate migration projections were obtained from the NASA Socioeconomic Data and Applications Center (SEDAC), which provides estimates of bilateral migration (https://doi.org/10.7927/4jb8-e177) and internal migration (https://doi.org/10.7927/5tv3-ff20). Projected population distributions were obtained from figshare (https://doi.org/10.6084/m9.figshare.19608594.v2). Dengue transmission potential datasets are available at the CLIMADE project’s GitHub repository (https://github.com/CERI-KRISP/Dengue_Incidence_Prediction).

## Code availability

All data processing, analyses, and figure generation were performed using RStudio (version 4.5.0; 2025-04-11). All scripts used in this study are available at: https://github.com/CERI-KRISP/Climate_Migration_Dengue_Exposure.
